# Safety and Efficacy of Pancreaticoduodenectomy in Octogenarians

**DOI:** 10.3389/fsurg.2021.617286

**Published:** 2021-02-02

**Authors:** Yeqian Huang, Ramesh Damodaran Prabha, Terence C. Chua, Jennifer Arena, Krishna Kotecha, Anubhav Mittal, Anthony J. Gill, Jaswinder S. Samra

**Affiliations:** ^1^Department of Gastrointestinal Surgery, Royal North Shore Hospital, St Leonards, NSW, Australia; ^2^Northern Clinical School, University of Sydney, Sydney, NSW, Australia; ^3^South Western Clinical School, University of New South Wales, Sydney, NSW, Australia; ^4^Department of Surgery, QE II Jubilee Hospital, Metro South Health, Brisbane, QLD, Australia; ^5^School of Medicine, Griffith University, Gold Coast, QLD, Australia; ^6^Discipline of Surgery, The University of Queensland, Brisbane, QLD, Australia; ^7^Cancer Diagnosis and Pathology Group, Kolling Institute of Medical Research, St Leonards, NSW, Australia; ^8^Deparment of Anatomical Pathology, Royal North Shore Hospital, St Leonards, NSW, Australia; ^9^Macquarie University Hospital, Macquarie University, Sydney, NSW, Australia

**Keywords:** whipple, pancreaticodoudenectomy, octogenarian, older, elderly, pancreatic adenocarcinoma

## Abstract

**Backgrounds:** Pancreaticoduodenectomy (PD) remains the only hope of a cure in selected patients with pancreatic adenocarcinoma (PAC). With an aging population, there will be an increasing number of very elderly patients being diagnosed with PAC of whom a selected proportion would be suitable for PD. However, the literature on outcomes of elderly patients after PD remains ambiguous. Therefore, the aim of this study was to examine the safety and efficacy of PD in octogenarians with PAC.

**Methods:** A retrospective analysis of 304 patients with PAC undergoing PD. Patients were divided into two age groups using age of 80 years old as the cut-off.

**Results:** Overall mortality and major morbidity rates were 0.5 and 18.5%, respectively. The octogenarian group had a higher rate of mortality (6.3%, *n* = 1, *p* < 0.001), a higher rate of major morbidity (37.5%, *n* = 6, *p* = 0.042) and a longer hospital stay (*p* = 0.035). However, median survival of octogenarians was 15.6 months. Multivariate analysis showed age was not identified as a prognostic factor for major morbidity and overall survival.

**Conclusion:** Age alone should not be an exclusion criterion for consideration of PD. With careful selection, PD can be safely performed in octogenarians. Elderly patients should be referred to a specialized unit for an objective assessment to determine the suitability for this aggressive but potential curative approach.

## Introduction

Pancreatic adenocarcinoma (PAC) remains one of the leading causes of cancer reflected death worldwide and contributes to 6% of all cancer deaths. Treatment options of this disease include surgery, radiotherapy, and chemotherapy (1). Surgery in the form of pancreaticoduodenectomy (PD) remains the only hope of cure in selected patients with PAC of the head of pancreas. The inherent morbidity relating to surgery is significant with major complication rates occurring in up to 40% of patients and mortality rate ranging from 2 to 5% (2).

In Australia, it is estimated that the elderly population (i.e., over 65 years of age) will continue to increase in the coming years from 15.5% in 2015 to 22.5% in 2050. Additionally, the very elderly population (i.e., 85 years or above) is also expected to nearly double from 4.1% in 2015 to 8.1% in 2050 (3). The rapid rise in the number of the elderly can be attributed to the advancement of medical care, public health interventions and aging of the baby boomers (4, 5). With an aging population, there will be an increasing number of very elderly patients being diagnosed with pancreatic cancer of whom a selected proportion would be suitable for PD (4).

Perioperative outcomes of the elderly patients after PD reported in the literature have not been well-defined. Some studies suggested that advanced age is a risk factor for a higher rate of postoperative complications and mortality (6, 7), whereas others were not able to demonstrate an increased risk in elderly patients (8–10). The significant risk of major morbidity and mortality after PD would be more profound in the elderly due to diminished physiological reserve impacting their ability to withstand a major operation, ability to recover and subsequent functional outcomes. However, with improved techniques and perioperative care, the mortality associated with this procedure has significantly decreased, ranging from 2–5% in the literature (1). Thus, PD may be a feasible option in selected elderly patients in the current environment. Therefore, the aim of this study was to examine and compare the short-term and long-term outcomes of octogenarians and non-octogenarian patients with pancreatic adenocarcinoma who underwent PD to determine the safety and efficacy of performing this procedure in the elderly population in Australia.

## Materials and Methods

### Patient Selection

This is a retrospective review of prospectively collected data of patients with PAC who underwent PD at the Department of Upper Gastrointestinal Surgery, North Shore campus of University of Sydney between January 2004 and June 2019. Patients with stage IV disease were excluded. Informed consent was obtained from patients prior to entering clinical details to database. Patients were divided into two groups based on their age (<80 years old vs. ≥80 years old). The distribution of patients undergoing PD was calculated based on a five-year interval apart from the last period which included five and half years (i.e., 2004–2008 inclusive; 2009–2013 inclusive; 2014–2019 inclusive).

### Preoperative Assessment

All patients were assessed clinically by history and physical examination. This was followed by investigations including routine laboratory tests, a serum carbohydrate antigen 19.9 level in patients with suspected pancreatic adenocarcinoma, computed tomography or magnetic resonance imaging, and endoscopic ultrasonography with fine-needle aspiration in selected patients. All patients were discussed at a multidisciplinary team (MDT) meeting before and after surgery. Staging laparoscopy and peritoneal lavage were performed prior to PD where indicated by the MDT.

### Surgical Technique

Standardized PD was performed with modified extended lymphadenectomy as previously described (11). Vascular resection was performed where indicated and technical aspects have been previously reported (12).

### Data Collection

The following data were retrieved for each patient: demographics including age, sex, American Society of Anesthesiologist (ASA) physical status classification; tumor characteristics including tumor size, margin clearance, lymph node involvement, stage and neurovascular invasion; perioperative factors including vascular resection, duration of surgery, blood loss and use of perioperative transfusion; postoperative outcomes including length of hospital stay, mortality, morbidity grade based on Clavien-Dindo classification (13). Surgical pathology was reported by an experienced gastrointestinal pathologist using a structured reporting protocol (14).

### Statistical Analysis

Statistical analyses were performed using SPSS for Windows version 24 (IBM Corporation, New York, USA). Continuous data were expressed as means and standard deviations (SD) or medians and ranges. Comparison of continuous variables was performed using independent *t* test. Categorical variables were analyzed using the Chi-square test or Fisher' exact test where appropriate. Multivariate analysis of risk factors for major morbidity was performed using a binary logistic regression model. Factors with a *P*-value of <0.2 in univariate analysis were included in multivariate analysis using logistic regression. Survival analysis was performed using the Kaplan-Meier curves and Log Rank test. A significant difference was defined as *P* < 0.05.

## Results

### Patient Demographics

**Figure 1:**
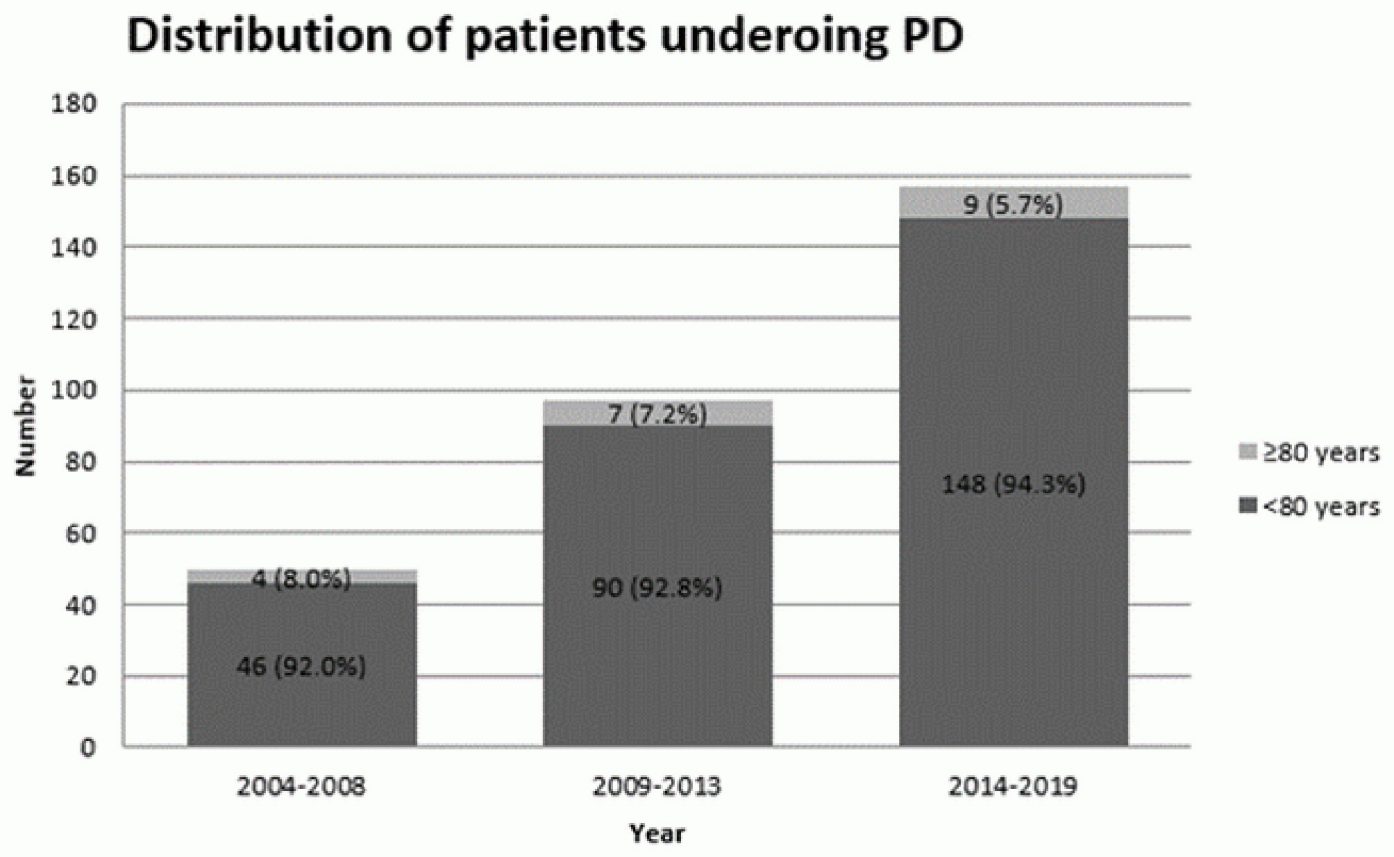
Distribution of patients undergoing PD.

A total of 304 patients were included in this study. Characteristics of the study cohort are shown in Table 1. There were 284 patients (93.4%) in the non-octogenarian group and 20 patients (6.6%) in the octogenarian group. The mean age was 66.1 years old (Standard Deviation (SD) = 9.5, Median = 67.0, Range = 32.0–85.0). There was a significant difference in the mean age in two age groups (*P* < 0.001). Ninety seven patients (33.4%) were diagnosed with severe systemic disease prior to PD (i.e., ASA≥3). There was a higher incidence of severe systematic disease in the very elderly group as compared to other patients (*P* < 0.001). Despite that the absolute number of octogenarians who underwent PD slightly increased over the years, the actual percentage of octogenarian patients who underwent PD were decreased in the recent years (Figure 1).

**Table 1:** Demographics.

	**Total**	** <80 years**	**≥80 years**	***P***
***N*** **(%)**	304	284 (93.4)	20 (6.6)	
**Sex** ***n*** **(%)**				*0.606*
Male	165 (54.5)	153 (54.1)	12 (60.0)	
Female	138 (45.5)	130 (45.9)	8 (40.0)	
**Age (years)**				
Mean ± SD	66.1 ± 9.5	65.0 ± 8.9	81.7 ± 1.7	* <0.001*
Median	67.0	66.5	81.5	
Range	32.0–85.0	32.0–79.0	80.0–85.0	
**ASA score** ***n*** **(%)**				* <0.001*
1/2	193 (66.6)	187 (69.3)	6 (30.0)	
3/4/5	97 (33.4)	83 (30.7)	14 (70.0)	
**Tumor characteristics**				
**Diameter (mm)**				
Mean ± SD	33.6 ± 14.0	33.6 ± 13.8	33.5 ± 17.2	*0.954*
Median	32.0	32.0	34.0	
Range	4.0–100.0	4.0–100.0	11.0–90.0	
Margins				*0.764*
R0	84 (27.9)	79 (28.1)	5 (25.0)	
R1	217 (72.1)	202 (71.9)	15 (75.0)	
Nodes				*0.282*
Positive	202 (67.8)	187 (67.0)	15 (78.9)	
Negative	96 (32.2)	92 (33.0)	4 (21.1)	
Stage				*0.579*
I	23 (7.7)	21 (7.5)	2 (10.5)	
II	238 (79.9)	222 (79.6)	16 (84.2)	
III	37 (12.4)	36 (12.9)	1 (5.3)	
Vascular invasion *n* (%)				*0.073*
Yes	165 (55.7)	150 (54.3)	15 (75.0)	
No	131 (44.3)	126 (45.7)	5 (25.0)	
Perineural invasion (%)				*0.510*
Yes	219 (73.7)	203 (73.3)	16 (80.0)	
No	78 (26.3)	74 (26.7)	4 (20.0)	

*SD, standard deviation; ASA, American Society of Anaesthesiologists*.

Table 1 provides a summary of the pathological features. The mean tumor size was 33.6 mm (SD = 14.0, Median = 32.0, Range = 4.0–100.0). 72.1% of patients (*n* = 217) had a R1 resection, whereas 67.8% of patients (*n* = 202) had positive lymph node involvements. The details of different stages in this study cohort were as follows: 23 patients (7.7%) with stage I disease; 238 patients (79.9%) with stage II disease; 37 patients (12.4%) with stage III disease. There were no statistical differences in mean tumor diameter (*P* = 0.954), R0 resection rate (*p* = 0.764), the incidence of lymph node involvement (*P* = 0.282), stage of the disease (*p* = 0.579), vascular invasion (*P* = 0.073) and perineural invasion (*P* = 0.510) between the two age groups.

### Perioperative Factors

The details of perioperative outcomes are provided in Table 2. The mean operative time and intraoperative blood loss were 418.0 min (SD = 106.1. Median = 410.0, Range = 190.0–970.0) and 584.5 ml (SD = 500.0, Median = 500.0, Range = 30.0–6500.0) respectively. Thirty nine patients (17.8%) required a perioperative transfusion. The mean units of packed red blood cells (PRBCs) required for perioperative was 3.4 (SD = 4.2, Median = 2.0, Range = 1.0–27.0). 80.1% of patients (*n* = 165) also had a vein resection. There was a significantly shorter mean operative time (*P* = 0.003). However, no statistical variation was demonstrated between the two study groups in terms of intraoperative blood loss (*P* = 0.895), use of perioperative transfusion (*P* = 0.193), mean perioperative transfusion units (*P* = 0.662) and vein resection rate (*P* = 0.767).

**Table 2:** Perioperative factors.

	**Total**	** <80 years**	**≥80 years**	***P***
***N*** **(%)**	304	284 (93.4)	20 (6.6)	
**Operative time (min)**				
Mean ± SD	418.0 ± 106.1	423.1 ± 105.9	349.8 ± 84.5	*0.003*
Median	410.0	410.0	335.0	
Range	190.0–970.0	230.0–970.0	190.0–500.0	
**Intraoperative blood loss (ml)**				
Mean ± SD	584.5 ± 543.6	585.6 ± 546.9	569.0 ± 507.6	*0.895*
Median	500.0	500.0	475.0	
Range	30.0–6,500.0	30.0–6,500.0	90.0–2,400.0	
Perioperative transfusion *n* (%)				*0.193*
Yes	39 (17.8)	34 (16.8)	5 (29.4)	
No	180 (82.2)	168 (83.2)	12 (70.6)	
**Perioperative transfusion (units of PRBCs)**				
Mean ± SD	3.4 ± 4.2	3.5 ± 4.5	2.6 ± 1.3	*0.662*
Median	2.0	2.0	2.0	
Range	1.0–27.0	1.0–27.0	1.0–4.0	
**Vein resection (%)**				*0.767*
Yes	165 (80.1)	155 (80.3)	10 (76.9)	
No	41 (19.9)	38 (19.7)	3 (23.1)	

### Postoperative Outcomes

The details of postoperative outcomes of this study cohort are outlined in Table 3. The overall mortality was 0.5%. There was one patient in octogenarian group who died during the admission, resulting in a difference in mortality rate between two groups (*P* < 0.001). There was a significantly higher major morbidity rate in octogenarian group (*P* = 0.042). Thirty four patients (15.5%) had a postoperative transfusion. The mean units of PRBCs required for postoperative transfusion was 2.7 (SD = 2.4, Median = 2.0, Range = 1.0–14.0). The mean length of hospital stay (LOS) was 17.3 days (SD = 12.6, Median = 14.0, Range = 6.0–156.0). The incidences of postoperative transfusion and mean units of postoperative transfusion was similar between two groups (*P* = 0.343 and *P* = 0.695 respectively).

**Table 3:** Postoperative outcomes.

	**Total**	** <80 years**	**≥80 years**	***P***
*N* (%)	304	284 (93.4)	20 (6.6)	
Mortality *n* (%)	1 (0.5)	0 (0)	1 (6.3)	* <0.001*
Major morbidity (i.e., Grade 3/4/5)	40 (18.5)	34 (17.0)	6 (37.5)	*0.042*
**Postoperative transfusion** ***n*** **(%)**				*0.343*
Yes	34 (15.5)	30 (14.9)	4 (23.5)	
No	185 (84.5)	172 (85.1)	13 (76.5)	
**Postoperative transfusion (units of PRBCs)**				
Mean ± SD	2.7 ± 2.4	2.8 ± 2.5	2.3 ± 1.0	*0.695*
Median	2.0	2.0	2.5	
Range	1.0–14.0	1.0–14.0	1.0–3.0	
**Postoperative length of stay (days)**				
Mean ± SD	17.3 ± 12.6	16.9 ± 12.3	23.0 ± 14.5	*0.035*
Median	14.0	14.0	18.5	
Range	6.0–156.0	6.0–156.0	10.0–64.0	
Median survival (months, 95%CI)	22.2!!break (18.9–25.5)	22.7!!break (20.2–−25.3)	15.6!!break (7.3–23.9)	*0.040*
1-year OS (%)	72.5	73.6	58.8	
3-year OS (%)	28.5	30.1	11.8	
5-year OS (%)	14.7	15.7	5.9	

The results for prognostic factor analysis are summarized in Table 4. Univariate and multivariate analysis did not identify age ≥80 as a prognostic factor for major morbidity. It was found that increasing length of stay was associated with a higher rate of major morbidity (OR = 1.15, 95% CI = 1.09–1.2, *P* < 0.001).

**Table 4:** Univariate and multivariate analysis of prognostic factors for major morbidity.

	**Univariate** ** (OR, 95%CI)**		**Multivariate** ** (OR, 95%CI)**	***P***
Age ≥ 80	2.93 (1.00–8.60)^*^	0.051	1.26 (0.32–4.96)	*0.742*
ASA ≥ 3	1.80 (0.89–3.62)^*^	0.100	1.29 (0.57–2.89)	*0.554*
Positive lymph nodes	0.95 (0.45–2.02)	0.896	-	
Operative time	1.00 (1.00–1.00)	0.960	-	
Perioperative transfusion	1.03 (0.42–2.55)	0.945	-	
Postoperative transfusion	3.55 (1.59–7.94)^*^	0.002	1.87 (0.73–4.81)	*0.194*
Vascular invasion	1.62 (0.77–3.41)	0.204	-	
LOS	1.15 (1.09–1.22)^*^	<0.001	1.15 (1.09–1.22)	* <0.001*

* *means p<0.2,OR>1 indicating more patients with major morbidity*.

### Survival Outcomes

**Figure 2:**
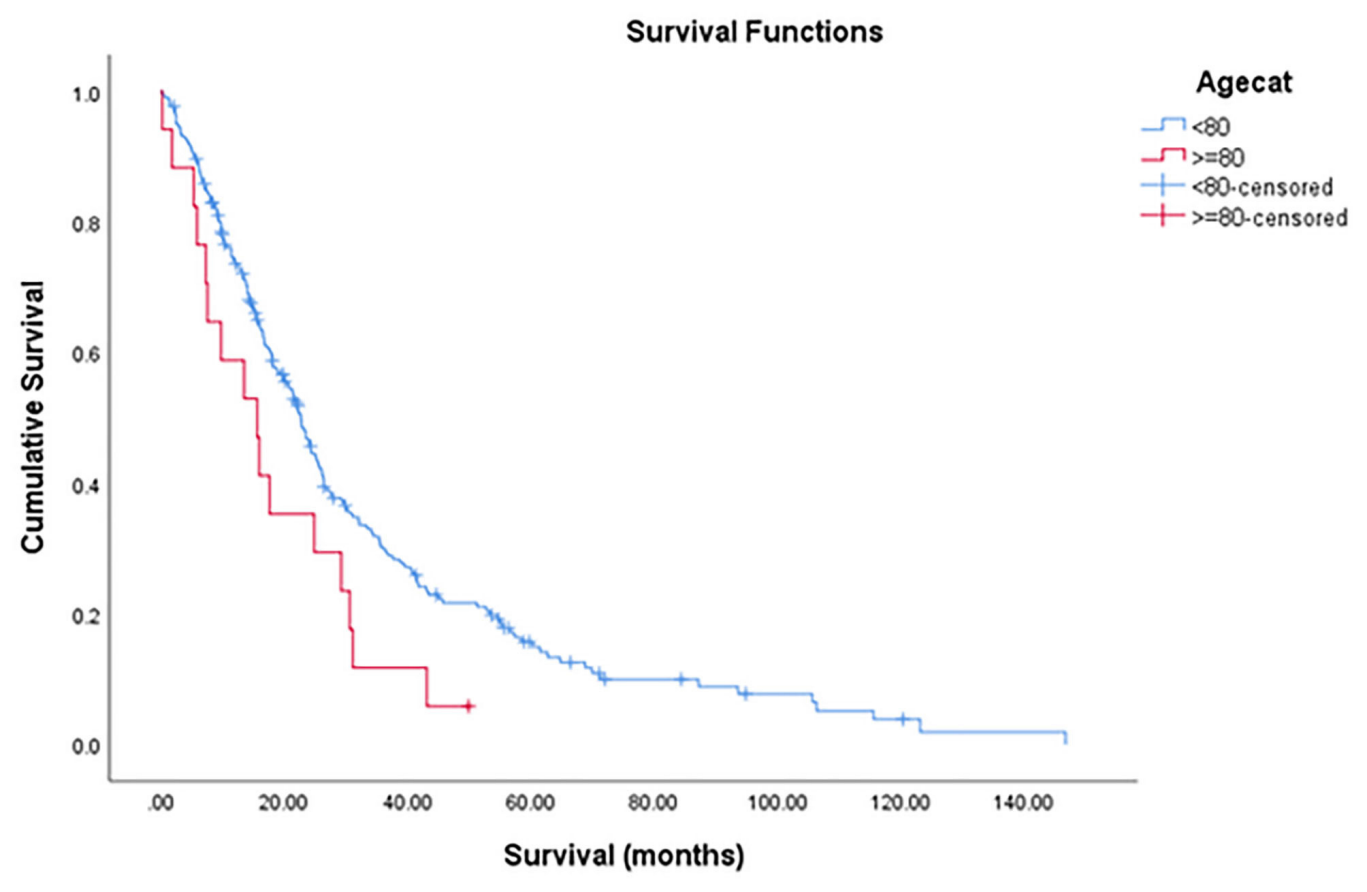
Overall survival (blue line <80 years old; Red line 80 years of above).

The median OS was 22.2 months (95% CI = 18.9–25.5) with a 1-year, 3-year, and 5-year OS rate of 72.5, 28.5, and 14.7% (Figure 2). The median OS was 22.7 months (95%CI = 20.2–25.3) for non-octogenarian group with a 1-year, 3-year, and 5-year OS rate of 73.6, 30.1, and 15.7%, whereas octogenarians had a median OS of 15.6 months (95%CI = 7.3–23.9) with a 1-year, 3-year and 5-year OS rate of 58.8, 11.8, and 5.9% (Table 3). In multivariate analysis, age was not a significant predictor for overall survival (*P* = 0.092) (Table 5).

**Table 5:** Univariate and multivariate analysis of prognostic factors for survival.

	**Univariate** ** (HR, 95%CI)**		**Multivariate** ** (HR, 95%CI)**	***P***
Age ≥ 80	1.71 (1.02–2.86)	0.042	1.19 (0.68–2.08)	*0.553*
ASA ≥ 3	1.15 (0.85–1.56)	0.378	-	
Positive lymph nodes	1.20 (1.40–2.83)^*^	<0.001	1.70 (1.11–2.60)	*0.015*
Operative time	1.00 (1.00–1.00)	0.248	-	
Perioperative transfusion	1.26 (0.85–1.87)	0.249	-	
Postoperative transfusion	1.33 (0.87–2.03)^*^	0.183	1/06 (0.67–1.68)	*0.802*
Vascular invasion	1.97 (1.43–2.70)^*^	<0.001	1.78 (1.22–2.59)	*0.003*
Major morbidity (Grade 3/4/5)	1.15 (0.85–1.56)^*^	0.079	1.38 (0.92–2.08)	*0.118*
LOS	1.02 (1.00–1.03)^*^	0.048	1.00 (0.971.02)	*0.846*

* *means p<0.2,OR > 1 indicating shorter survival*.

## Discussion

Considering PD for octogenarians requires a fine balance between frailty of the patient and benefits that surgery can potentially offer. The number of octogenarians remained similar in the last decade in our center despite increasing overall number of patients with PAC who underwent PD and improvement in surgical technique and perioperative care. It could highlight the possible barriers for elderly patients to surgery in the current context, including potential selection bias in referral process for eligible patients. Elderly patients are at risk of under-treatment due to the absence of clear guidelines and concerns about their tolerability of treatment (15, 16). Therefore, it is important to determine the role of age in the short-term and long-term outcomes after PD.

The preoperative decision-making process is complicated by limited data available on the very elderly patients with PAC undergoing PD. Kim et al. (17) conducted a systematic review and meta-analysis on outcomes of patients aged 80 years or over who underwent PD for treatment of pancreatic malignancy (17). They showed its 30-day mortality was twice that of the central group (under the age of 80 years old) and its risk of complications were increased by 50%. However, their study population is slightly different as they also included the studies on mixed benign and malignant pathologies. The threshold to perform PD in an octogenarian for non-PAC could be much higher.

Several recent studies have reported a mortality rate of <5% for those undergoing PD (4, 9, 18, 19). Our literature review has identified a median mortality rate of 4.0% (range = 2.0–4.4) for patients with PAC undergoing PD (Appendix Table 1). From our data, octogenarians have a postoperative mortality rate of 6.3%, which is albeit at the higher end, remains statistically comparable to their non-octogenarian counterparts (0). Despite the mortality rate, the actual number of deaths in octogenarian group was one. The patient was operated in 2012. Operative techniques and perioperative care in the earlier days should be considered. It is still at the midrange of the mortality rate reported by the systematic review conducted by Kim et al. (17), which ranges from 0–11%. One of the largest studies in the literature was conducted by de la Fuente et al. (6), who assessed clinical data of 6,293 patients, of which 593 patients were octogenarians (6). They reported similar higher mortality rates and serious complication rate in octogenarians as compared to findings in our study.

Our data demonstrate a significantly shorter operative time in the octogenarian patients with comparable intraoperative blood loss. This might be contributed by the fact that non-octogenarian patients tend to receive a more aggressive resection requiring a relatively longer operation. This could be demonstrated by the larger tumors resected in the non-octogenarian group. Our findings suggest a higher major morbidity rate with a longer hospital stay in the octogenarian group. It can be contributed by the fact that elderly populations are more likely to have a reduced physiologic reserve and other medical comorbidities (15, 17). Chronologic age is a poor indicator of physical, mental or medical functional status (19, 20). However, after considering other potential confounding factors, our logistic regression also illustrates age is not an independent prognostic factor for major morbidity after PD.

In this study, median survival of elderly patients was 15.6, which was consistent with the literature. Median survival in Octogenarian group varies from 11.6 months to 17.3 months from our literature search (Appendix Table 1). Therefore, surgery could still provide benefits for highly selected elderly patients. They are likely to have long-term survival benefits if they survive the initial postoperative period. Appropriate patient selection is essential to reduce perioperative morbidity and mortality for elderly patients with PAC following PD.

Therefore efforts should be made to develop an objective assessment of individual patients which can assist in tailoring treatment, improving outcomes and reducing complications. A novel approach of integrating sarcopenia with self-reported exhaustion has been proved to be accurate in identify frail elderly patients undergoing PD (21).

In addition, optimizing postoperative care is also crucial. Long-term survival of these patients is mainly dependent on early mortality. Those who survive the first year after surgery have the similar cancer-related survival as younger patients (15, 22). Medical staff should be educated about recognizing specific geriatric complications including delirium, communication with hearing impaired patients or those with cognitive impairments, and managing their co-morbidity and polypharmacy (16). Elderly patients typically use multiple medications that frequently need to be continued immediately postoperatively (6).

Over the last few years, there has been emerging evidence of the benefits of laparoscopic PD (LPD) in the elderly patients (10, 23–26). Although the initial concern is the oncological outcomes, there have been some evidence on the oncological safety of LPD (23, 25). It was suggest to be associated with less intraoperative blood loss, shorter hospital stay (10, 24) and lower 90-day mortality (23) in the elderly population without compromising the survival benefits (25). However, the lack of sufficient training courses or programs is a barrier in the introduction of LPD (27). With more promising data and introduction of more training, LDP could be an appealing approach for elderly patients with PAD.

To our knowledge, this is the first Australian series on PD for octogenarians. Several limitations need to be considered when interpreting the results of this study. It is limited by the small sample size of octogenarians and retrospective nature which could lead to selection bias. Also, perioperative outcomes of PD heavily depend upon the experience of attending surgeon and institutional volume. This study was conducted in a specialized unit in Australia. Octogenarian surgery also tends to only happen in first world country due to longevity of living. Thus, survival is likely also dependent on the country itself and their life expectancy In addition, other factors that could potentially affect patient outcomes including preoperative nutritional status, cachexia, neo-adjuvant or adjuvant therapy were not included in this study (7). The percentage of patients who are aged 80 or above and underwent PD were decreasing in last few years at our center. It is thus important to consider referral bias. The number of elderly patients who were not referred for evaluation and turned down based on their age alone was unclear.

## Conclusion

Age alone should not be an exclusion criterion for consideration of PD. Patients who are 80 years and older should be referred to a specialized unit for an objective assessment to determine the suitability for this aggressive but potential curative approach. Elderly patients when carefully selected can undergo PD safely with beneficial survival outcomes. Also, more education about managing geriatric patients postoperatively should be delivered to medical staff to optimize postoperative care for these patients. More Quality of Life studies with a larger sample size of Octogenarians are warranted to determine the survivorship benefits.

## Data Availability Statement

The data analyzed in this study is subject to the following licenses/restrictions: It is used for research purpose only. Requests to access these datasets should be directed to Professor Jaswinder Samra, jas.samra@bigpond.com.

## Ethics Statement

Ethical review and approval was not required for the study on human participants in accordance with the local legislation and institutional requirements. The patients/participants provided their written informed consent to participate in this study.

## Author Contributions

YH, RD, TC, JA, KK, AM, AG, and JS: study concepts and study design. RD, JS, and JA: data acquisition. YH, RD, TC, JA, KK, AM, AG, and JS: quality control of data and algorithms. YH, TC, and JS: data analysis and interpretation. YH: statistical analysis and manuscript preparation. YH, RD, TC, KK, AM, AG, and JS: manuscript editing. YH, RD, TC, JA, KK, AM, AG, and JS: manuscript review. All authors contributed to the article and approved the submitted version.

## Conflict of Interest

The authors declare that the research was conducted in the absence of any commercial or financial relationships that could be construed as a potential conflict of interest.
