# Supplementary material for: Safety and Efficacy of Pancreaticoduodenectomy in Octogenarians
Source: Front Surg. 2021 Feb 2;8:617286. doi: 10.3389/fsurg.2021.617286 (PMC7884922; doi:10.3389/fsurg.2021.617286)
Supplement: Supplementary file 1 [file Table_1.docx]

Appendix Table 1 Summary of literature on outcomes of patients aged 80 years or above

| Author | Country | Study Period | Study | Total number of patients | Median Survival (Months) | Mortality % (n) | Morbidity % (n) |
| --- | --- | --- | --- | --- | --- | --- | --- |
| Sohn et al. (1998)(1) | US | Dec 1986-Jun 1996 | Mixed- benign and malignant | 46 | 38.0 | 4.3 (2) | 57.0 (26) |
| Makary et al. (2006)(2) | US | Apr 1970-Mar 2005 | Mixed malignancies | 207 | NR | 3.5 (8) | 52.6 (109) |
| Tani et al. (2009)(3) | Japan | Jan 1994-Aug 2008 | Mixed- benign and malignant | 25 | NR | 0 (0) | NR |
| Khan et al. (2010)(4) | US | 1981-2007 | PAC | 53 | 13.5 | 2 (1) | 51 (27) |
| Lee et al. (2010) (5) | US | Feb 1992-Dec 2009 | PAC | 45 | 11.6 | 4.4 (2) | 48.9 (22) |
|  |  |  | Mixed-benign and malignant | 74 | NR | 5.4 (4) | 47.3 (35) |
| De La Fuente et al. (2011)(6) | US | 2005-2009 | NR | 593 | NR | 6.3 (37) | 45.2 (268) |
| Hatzaras et al. (2011) (7) | US | Jan 1990-Dec 2007 | Mixed malignancies | 27 | 33.3 | 3.7 (1) | 52.0 (14) |
| Melis et al. (2012)(8) | US | 1990-2009 | PAC | 25 | 17.3 | 4 (1) | 44.0(77) |
| Oguro et al. (2013)(9) | US | Jan 2001-Dec 2009 | Mixed- benign and malignant | 22 | 13.0 (for PAC) | 4.5 (10) | - |
| Belyaev et al. (2013)(10) | Germany | Jan 2004-Dec 2010 | Mixed- benign and malignant | 76 | 19.0(for malignant lesion) | 11.8 (9) | 72.4 (55) |
| Gangl et al. (2014)(11) | Austria | 2001-2010 | Pancreatic ductal adenocarcinoma | 6 | 10.5 | 0 (0) | 28.3 (2) |
| Lee et al. (2014)(12) | US | 2005-2010 | NR | 475 | NR | 6.0 (29) | NR |
| Beltrame et al. (2015)(13) | Italy | Jan 1998- Dec 2011 | Mixed- benign and malignant | 23 | 19.0 (for PAC) | 0 (0) | 43.5 (10) |
| Langan et al. (2015)(14) | US | 2010-2014 | NR | 900 | NR | 6.3 (57) | 54.7 (492) |
| Liang etl al. (2016)(15) | US | 2007-2015 | Mixed- benign and malignant | 33 | NR | 3.0 (1) | 27.3 (9) |
| Langan et al. (2016)(16) | US | 2005-2013 | Mixed- benign and malignant | 33 | NR | NR | 51.5 (17) |
| Total number | - | - | - | 2618 | - | - | - |
| Median | - | - | - | - | 13.5 (for PAC only)  17.3 (overall) | 4.0 (for PAC only)  4.0 | 48.9 (for PAC only)  51.0 (overall) |
| Range | - | - | - | - | 11.6-17.3 (for PAC only)  10.5-38.0 (overall) | 2.0-4.4 (for PAC only)  0-11.8 (overall) | 27.3- 72.4 (for PAC only)  44.0-51.0 (overall) |

NR-Not reported, PAC- pancreatic adenocarcinoma

Reference

1. Sohn, T. A., Yeo, C. J., Cameron, J. L., Lillemoe, K. D., Talamini, M. A., Hruban, R. H., Sauter, P. K., Coleman, J., Ord, S. E., Grochow, L. B., Abrams, R. A. and Pitt, H. A.: (1998) Should pancreaticoduodenectomy be performed in octogenarians? *J Gastrointest Surg*, 2(3), 207-16 doi:10.1016/s1091-255x(98)80014-0

2. Makary, M. A., Winter, J. M., Cameron, J. L., Campbell, K. A., Chang, D., Cunningham, S. C., Riall, T. S. and Yeo, C. J.: (2006) Pancreaticoduodenectomy in the very elderly. *J Gastrointest Surg*, 10(3), 347-56 doi:10.1016/j.gassur.2005.12.014

3. Tani, M., Kawai, M., Hirono, S., Ina, S., Miyazawa, M., Nishioka, R., Shimizu, A., Uchiyama, K. and Yamaue, H.: (2009) A pancreaticoduodenectomy is acceptable for periampullary tumors in the elderly, even in patients over 80 years of age. *J Hepatobiliary Pancreat Surg*, 16(5), 675-80 doi:10.1007/s00534-009-0106-6

4. Khan, S., Sclabas, G., Lombardo, K. R., Sarr, M. G., Nagorney, D., Kendrick, M. L., Donohue, J. H., Que, F. G. and Farnell, M. B.: (2010) Pancreatoduodenectomy for ductal adenocarcinoma in the very elderly; is it safe and justified? *J Gastrointest Surg*, 14(11), 1826-31 doi:10.1007/s11605-010-1294-8

5. Lee, M. K., Dinorcia, J., Reavey, P. L., Holden, M. M., Genkinger, J. M., Lee, J. A., Schrope, B. A., Chabot, J. A. and Allendorf, J. D.: (2010) Pancreaticoduodenectomy can be performed safely in patients aged 80 years and older. *J Gastrointest Surg*, 14(11), 1838-46 doi:10.1007/s11605-010-1345-1

6. de la Fuente, S. G., Bennett, K. M., Pappas, T. N. and Scarborough, J. E.: (2011) Pre- and intraoperative variables affecting early outcomes in elderly patients undergoing pancreaticoduodenectomy. *HPB (Oxford)*, 13(12), 887-92 doi:10.1111/j.1477-2574.2011.00390.x

7. Hatzaras, I., Schmidt, C., Klemanski, D., Muscarella, P., Melvin, W. S., Ellison, E. C. and Bloomston, M.: (2011) Pancreatic resection in the octogenarian: a safe option for pancreatic malignancy. *J Am Coll Surg*, 212(3), 373-7 doi:10.1016/j.jamcollsurg.2010.10.015

8. Melis, M., Marcon, F., Masi, A., Pinna, A., Sarpel, U., Miller, G., Moore, H., Cohen, S., Berman, R., Pachter, H. L. and Newman, E.: (2012) The safety of a pancreaticoduodenectomy in patients older than 80 years: risk vs. benefits. *HPB (Oxford)*, 14(9), 583-8 doi:10.1111/j.1477-2574.2012.00484.x

9. Oguro, S., Shimada, K., Kishi, Y., Nara, S., Esaki, M. and Kosuge, T.: (2013) Perioperative and long-term outcomes after pancreaticoduodenectomy in elderly patients 80 years of age and older. *Langenbecks Arch Surg*, 398(4), 531-8 doi:10.1007/s00423-013-1072-7

10. Belyaev, O., Herzog, T., Kaya, G., Chromik, A. M., Meurer, K., Uhl, W. and Müller, C. A.: (2013) Pancreatic surgery in the very old: face to face with a challenge of the near future. *World J Surg*, 37(5), 1013-20 doi:10.1007/s00268-013-1944-6

11. Gangl, O., Fröschl, U. and Függer, R.: (2014) Surgical quality data and survival after pancreatic cancer resections: a comparison of results for octogenarians and younger patients. *Wien Klin Wochenschr*, 126(23-24), 757-61 doi:10.1007/s00508-014-0603-8

12. Lee, D. Y., Schwartz, J. A., Wexelman, B., Kirchoff, D., Yang, K. C. and Attiyeh, F.: (2014) Outcomes of pancreaticoduodenectomy for pancreatic malignancy in octogenarians: an American College of Surgeons National Surgical Quality Improvement Program analysis. *Am J Surg*, 207(4), 540-8 doi:10.1016/j.amjsurg.2013.07.042

13. Beltrame, V., Gruppo, M., Pastorelli, D., Pedrazzoli, S., Merigliano, S. and Sperti, C.: (2015) Outcome of pancreaticoduodenectomy in octogenarians: Single institution's experience and review of the literature. *J Visc Surg*, 152(5), 279-84 doi:10.1016/j.jviscsurg.2015.06.004

14. Langan, R. C., Zheng, C., Harris, K., Verstraete, R., Al-Refaie, W. B. and Johnson, L. B.: (2015) Hospital-level resource use by the oldest-old for pancreaticoduodenectomy at high-volume hospitals. *Surgery*, 158(2), 366-72 doi:10.1016/j.surg.2015.02.022

15. Liang, D. H., Shirkey, B. A., Rosenberg, W. R. and Martinez, S.: (2016) Clinical outcomes of pancreaticoduodenectomy in octogenarians: a surgeon's experience from 2007 to 2015. *J Gastrointest Oncol*, 7(4), 540-6 doi:10.21037/jgo.2016.03.04

16. Langan, R. C., Huang, C. C., Mao, W. R., Harris, K., Chapman, W., Fehring, C., Oza, K., Jackson, P. G., Jha, R., Haddad, N., Carroll, J., Hanna, J., Parker, A., Al-Refaie, W. B. and Johnson, L. B.: (2016) Pancreaticoduodenectomy hospital resource utilization in octogenarians. *Am J Surg*, 211(1), 70-5 doi:10.1016/j.amjsurg.2015.04.014
